# An Add‐On Contactless Measurement System for Monitoring Driving Behaviours in Motorised Mobility Scooters

**DOI:** 10.1049/htl2.70064

**Published:** 2026-02-11

**Authors:** Yi Liu, Takenobu Inoue, Jun Suzurikawa

**Affiliations:** ^1^ College of Mechanical and Electrical Engineering Harbin Engineering University Harbin China; ^2^ Department of Assistive Technology, Research Institute National Rehabilitation Center For Persons with Disabilities Tokorozawa Japan; ^3^ Yangtze Delta Region Advanced Research Institute of Harbin Engineering University NanTong China

**Keywords:** accelerometers, image motion analysis, motion measurement, patient rehabilitation, wheelchairs

## Abstract

The increasing use of motorised mobility scooters (MMSs) has raised significant safety concerns, particularly related to user behaviour during operation. Although various advanced driving assistance systems have been incorporated into MMSs to identify potential environmental hazards, few studies have investigated the impact of user behaviour in MMS driving. This study was the first to incorporate automated behaviour monitoring into the evaluation of MMS driving using an add‐on driving behaviour monitoring system (ADBMS). The ADBMS was a platform for contactless measurement that used a pre‐trained convolutional neural network to estimate posture and two inertial measurement units to record steering and throttle operations. Experiments were conducted to demonstrate the usability of the ADBMS and evaluate the coordination of head movements and steering manoeuvres when driving an MMS in the outdoor environment. Cross‐correlation analysis revealed that head movement consistently preceded steering operation during the driving tasks, indicating the potential of the proposed system to quantify user behaviour related to attention toward the surrounding environment. The lag time between these two parameters may serve as a novel index of driving safety. These findings could support a comprehensive understanding of users’ driving behaviours and provide valuable insights into developing behavioural interventions to promote safer MMS use.

## Graphical abstract

The increasing use of motorised mobility scooters (MMSs) has raised safety concerns related to user behaviour. We developed an easy‐to‐install, cost‐effective add‐on driving behaviour monitoring system (ADBMS) that estimates head orientation using a pre‐trained CNN and simultaneously records steering operations with IMUs. Outdoor experiments showed that head movement consistently precedes steering, and the cross‐correlation lag between them may serve as a novel safety index, offering insights for behavioural interventions to promote safer MMS use.

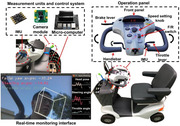

## 1 Introduction

Motorised mobility scooters (MMSs) have become a critical assistive technology, significantly enhancing the quality of life for older adults and individuals with mobility impairments [1]. As a practical alternative to walking, MMSs effectively extend users’ mobility capabilities, enabling them to participate more actively in a broader range of activities in local communities (e.g., a relaxing stroll, shopping, or hospital visit) [2, 3]. With the growth in the global ageing population, MMSs have been gaining popularity among older individuals because they are easy to acquire, and no driving licence is required. However, the widespread adoption of MMSs has raised concern regarding driving safety.

The number of MMS‐related accidents in Sweden increased threefold over the 10 years from 2007 to 2016, highlighting the severity of the safety issue [4]. In the same period, 69 people aged 60 and over died from MMS‐related incidents in Australia. Advanced driving assistance systems and obstacle detection technologies have been incorporated into MMSs to autonomously perceive the environment and identify potential hazards to improve MMS driving safety [5]. Despite these technological advancements, few studies have investigated the effects of a user's driving behaviour on MMS safety. Human factors account for a large portion of MMS‐related accidents [6]. Because MMS users are primarily older or mobility‐impaired individuals who experience a decline in sensory and cognitive functions, they easily omit the surrounding hazardous factors while driving, exposing them to the risk of injury [7, 8, 9]. Therefore, monitoring and evaluating the user's driving behaviour may contribute to improving the driving safety of MMSs.

Driving an MMS may be challenging for older individuals or those with impaired mobility because this process requires specific capabilities and skills, such as navigating the route, avoiding obstacles, and obeying traffic rules [10]. Some performance‐based assessment methods (e.g., power‐mobility community driving assessments) have been proposed to evaluate the individual's capability and skills during driving an MMS [11, 12]. However, these methods employ a laborious and qualitative assessment process primarily based on the visual observations of a human evaluator. The evaluation result, therefore, may be biased by the rater's experience and expertise.

A more promising and cost‐effective alternative is a sensor‐based system to monitor the individual user's driving behaviour and further evaluate the user's driving performance [13]. In our previous study, a kinematic model‐based method was proposed to estimate the operating angles of the steering tiller and throttle lever using the acceleration data of two inertial measurement units (IMUs) [14]. Based on this approach, a driving operation logging system was developed to monitor the user's driving operation and provide insights into how the user's driving behaviour changed over the long term [15]. Despite these efforts for driving operation analysis, the user's driving behaviour has not been thoroughly investigated because of the lack of user motion monitoring.

To address this gap, this study presented the first attempt to incorporate automated behaviour monitoring into the quantitative driving evaluation of MMSs utilising an add‐on driving behaviour monitoring system (ADBMS). The ADBMS was developed as an easy‐to‐install, cost‐effective platform for contactless measurement of the user's driving behaviour in MMSs. It used a pre‐trained convolutional neural network (CNN) to estimate the user's driving pose from the camera‐captured image sequences, which were fused with simultaneous steering manoeuvres to analyse driving behaviour comprehensively. With the ADBMS, we evaluated how the head movements and steering manoeuvres are coordinated during MMS driving and quantified the time lag between them using cross‐correlation analysis.

## 2 Methods

### 2.1 The ADBMS for Contactless Behaviour Monitoring in MMS Driving

**FIGURE 1:**
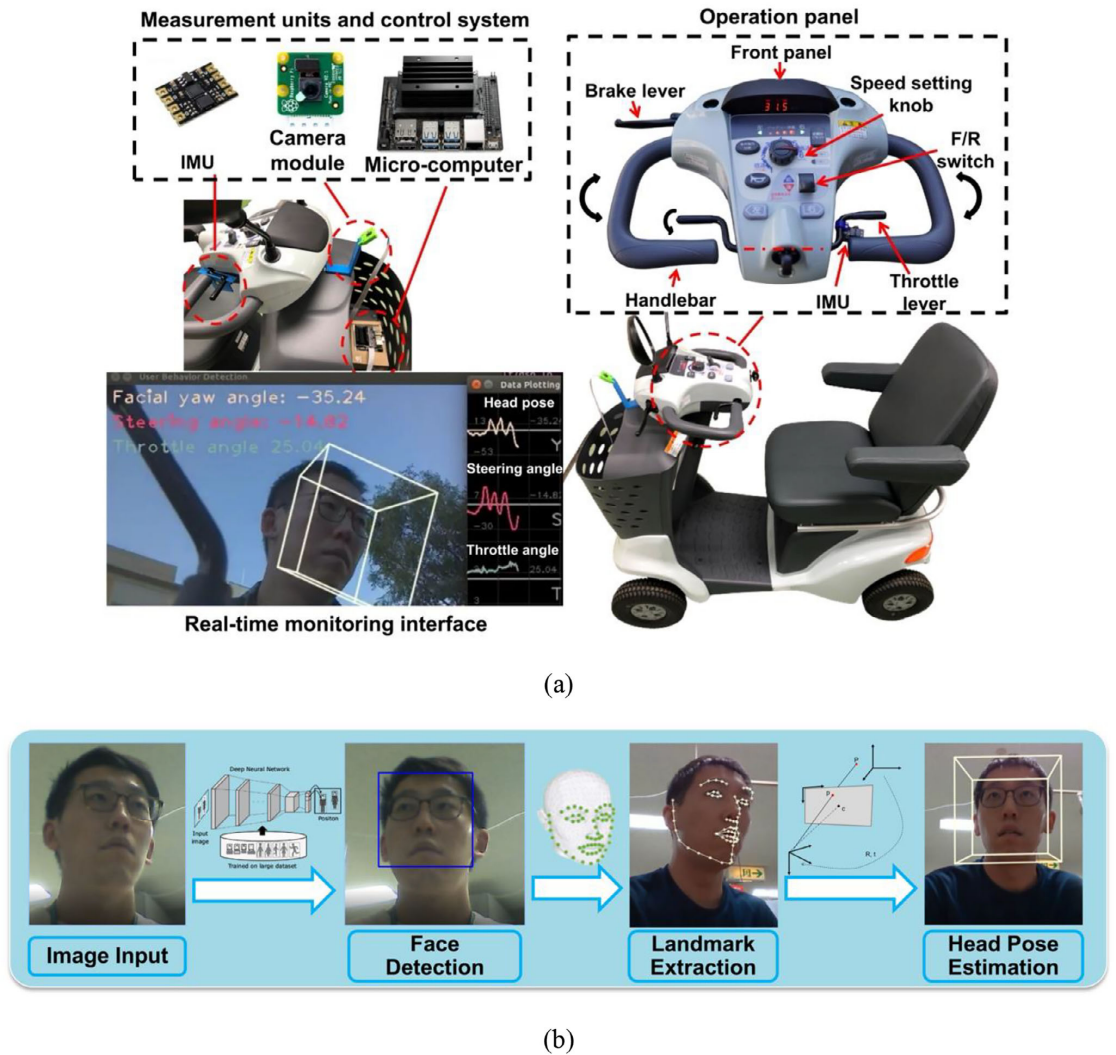
(a) Overview of the developed system. It mainly consisted of a microcomputer, a camera module and two IMUs. These measurement units and the control board were mounted on a four‐wheel MMS to real‐time monitor the user's driving behaviour. On the operation panel of the MMS, a handlebar was used for steering manoeuvres and a throttle lever was positioned close to the handlebar that can adjust the speed of the MMS. (b) Pipeline of the CNN‐based head pose estimation. First, the user's image input was fed to a lightweight CNN model for face detection. The image sequence was then used to extract the key facial landmarks and dynamically reconstruct a user‐specific 3D face model. Head pose was subsequently estimated using a solvePnP algorithm.

The ADBMS is a low‐cost and easy‐to‐install platform using multiple sensors to implement a contactless measurement of driving behaviour. Without any sensor attached to the driver, the proposed system can quantify and record steering and speed‐control operation and head orientation during driving, a helpful index that indicates the user's driving attention [16]. As depicted in Figure 1a, it consisted of a microcomputer (Jetson Nano, Nvidia, U.S.A.), a small‐size camera module (Raspberry Pi camera module v2), and two IMUs (GY‐25t). The measurement devices and control board were mounted on a commercially available MMS (ET4D, Suzuki Motor Corp., Japan), a four‐wheel electric‐powered vehicle. A handlebar was connected to the front wheels through a steering column. A throttle lever was positioned close to the handlebar that can adjust the speed of the MMS.

The camera was mounted on the front basket facing the user to capture the user's driving pose. A CNN‐based head pose estimation method was applied to monitor the user's head movement while driving an MMS [17]; the pipeline is depicted in Figure 1b. First, the MobileNet [18], a lightweight CNN model, was employed to detect the user's face in the images captured by the camera. This architecture employed depthwise separable convolutions to reduce the number of parameters and computation latency, enabling efficient real‐time inference on the embedded microcomputer system. The image sequence was then fed into the microcomputer to extract the key facial landmarks in real time using a pretrained model, 3DDFA_v2 [19], and dynamically reconstruct a user‐specific 3D face model. Head pose was subsequently estimated in real time using a solvePnP algorithm by directly regressing the parameters of the camera matrix [20]. For simultaneously monitoring the user's driving operations, an IMU was attached to the throttle lever to measure the angles of the steering wheel and throttle lever. Given the inclinations and vibrations on different terrains, a supplementary IMU was attached to the scooter body to implement angle compensation. Data fusion was performed by subtracting the inclination angles measured by the body‐mounted IMU from those of the lever‐mounted IMU. This differential measurement minimised the effect of environmental factors such as slope inclination and vehicle vibration, ensuring that only the user's input was captured.

### 2.2 Driving Behaviour Analysis

Driving an MMS is comprehensive and includes visually guided environment perception, path planning, and vehicle manoeuvring [21]. The user's head movement and steering operation are closely linked when driving on a winding road. Because of the inherent limitations of the human visual field, drivers tend to turn their heads just prior to steering to acquire additional visual information, determining the appropriate action to guarantee safe driving. Therefore, appropriate head‐steering coordination is essential for MMS driving safety.

A cross‐correlation analysis was implemented in this study to characterise the individual's head‐steering coordination while driving an MMS [22]. The cross‐correlation was used to identify temporal directionality between two time‐series signals, such as a leader‐follower relationship, and quantify the time lag between them. For assessing the more fine‐grained dynamics of head‐steering coordination throughout the process, a sliding window cross‐correlation analysis was implemented to compare two signals by moving a sliding window from one signal over the other signal and calculating how similar they are at each time, which is defined by

(1) $$\;R_{h,s}=\frac{\sum_{i=t-n+1}^{t}\;\left[\left(x_{h}^{i}-\bar{x_{h}}\right)\left(x_{s}^{i}-\bar{x_{s}}\right)\right]}{\sqrt{\sum_{i=t-n+1}^{t}\;{\left(x_{h}^{i}-\bar{x_{h}}\right)}^{2}\cdot \sum_{i=t-n+1}^{t}\;{\left(x_{s}^{i}-\bar{x_{s}}\right)}^{2}}}\;$$

(2) $$\;\bar{x_{h}}=\frac{1}{n}\;\sum_{i=t-n+1}^{t}x_{h},\;\bar{x_{s}}=\frac{1}{n}\;\sum_{i=t-n+1}^{t}x_{s}$$

where *x_h_* and *x_s_* are the head‐yaw angle and steering angle, *i* is the i^th^ sampling point in the time series, and *n* is the number of sample data points in the sliding window. The *R_h,s_* is a vector bundle of correlation coefficient values ranging from −1 to 1, a measure of the covariation in the two time‐series signals. The cross‐correlation value of 1 means the two signals are entirely related.

This study used a 20‐second sliding window to segment the time‐series signals into consecutive epochs. This window size was selected to cover multiple turning cycles within a single epoch, ensuring sufficient signal variation for robust cross‐correlation calculation while maintaining adequate temporal resolution to observe dynamic changes in driving behaviour. The cross‐correlation analysis was repeatedly implemented by shifting one second (step size) at a time to calculate the time lag in each epoch. The obtained rolling window cross‐correlogram illustrated the dynamic interaction of head movements and steering manoeuvres throughout the process, thereby visualising how the lag time varied over the different phases.

### 2.3 Driving Experiment

The experimental trials were conducted on an outdoor wheelchair training ground in the National Rehabilitation Center for Persons with Disabilities (NRCD). The experimental procedures were approved by the Institutional Review Board of NRCD (protocol number: 2023–074), and all methods were performed in accordance with the relevant guidelines and regulations. Three healthy participants (males, age: 23 years old, height: 174.8 ± 11.5 cm, body mass: 64.3 ± 4.3 kg) were involved in the experiment. The participants were recruited from the population at NRCD through word‐of‐mouth. The inclusion criteria were (1) healthy adults with no physical disabilities; (2) normal or corrected‐to‐normal vision; and (3) no prior experience with the specific MMS model used in this study to avoid learning effects. The exclusion criteria included (1) any history of musculoskeletal or neurological disorders that could affect driving performance and (2) a history of severe motion sickness. All participants provided written informed consent.

**FIGURE 2:**
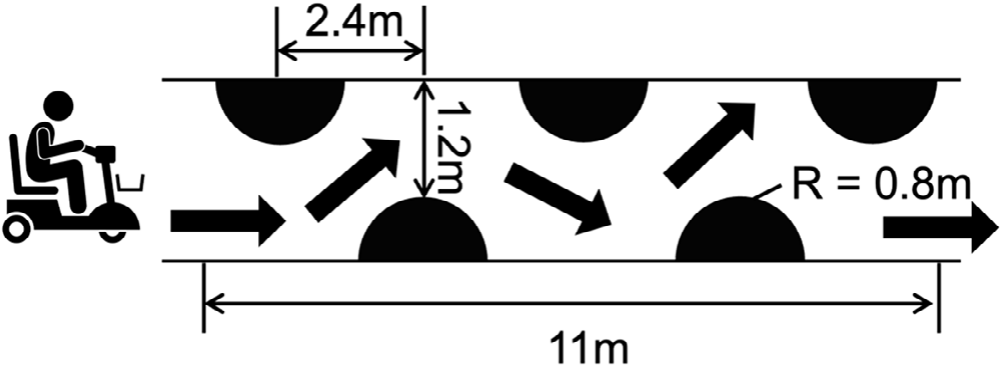
Slalom driving test. The participants were instructed to drive the MMS through a course of obstacles placed in a slalom pattern with an interval of 2.4 m and a path width of 1.2 m.

**FIGURE 3:**
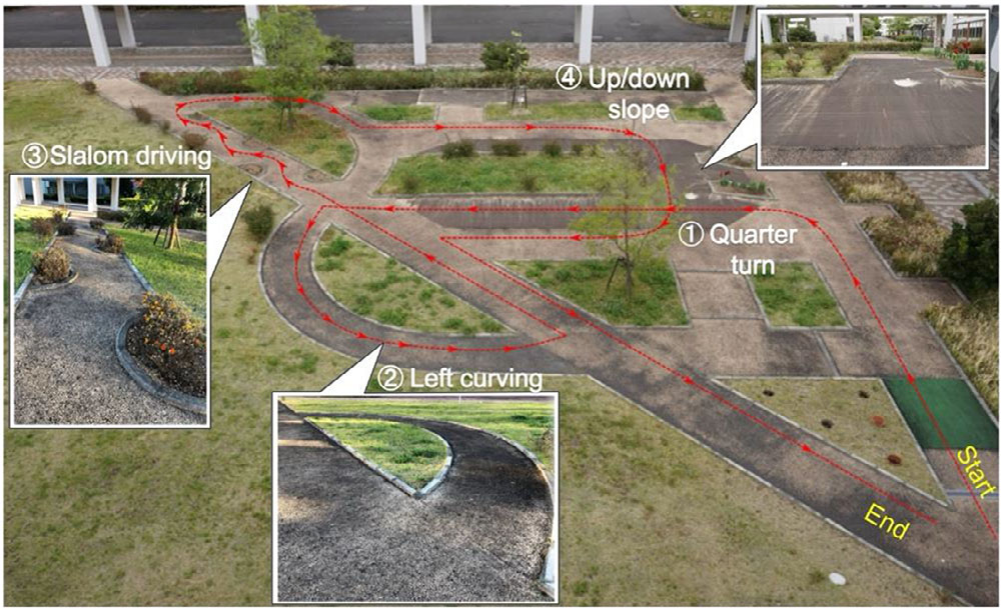
Driving courses for continuous measurement. (1) Quarter turn. (2) Left curving. (3) Slalom driving. (4) Up/down slope.

In demonstrating the usability of the ADBMS and evaluating the driving performance of each participant, a slalom driving test requiring frequent head turning and steering manoeuvres was first conducted to observe the user's behaviours while driving the MMS through a course of obstacles placed in a slalom pattern (Figure 2). A driving course simulating typical scenarios in daily driving of MMS was designed (Figure 3) to explore the head‐steering coordination and comprehensively analyse how the temporal relationship of head pose and steering operation varied over a period of continuous driving in the outdoor environment.

The participants were instructed to follow the designated path and complete the consecutive driving tasks for continuous measurement of the participant's driving behaviour. Four specified scenarios requiring close head‐steering coordination were segmented from the whole driving trial for a more fine‐grained analysis of the temporal relationship between head movements and steering manoeuvres: (1) quarter turn, (2) left curving, (3) slalom driving, and (4) up/down slope. After segmentation, a cross‐correlation analysis was conducted to quantify the head‐steering time lag in each task. The continuous driving trials were repeated five times to alleviate the effect of sudden changes associated with environmental factors. Throughout the experiment, the maximum speed of the MMS was set to 4 km/h.

### 2.4 Statistical Analysis

All statistical analyses were performed using MATLAB. Prior to correlation analysis, the Shapiro‐Wilk test was conducted to verify the normality assumption of the time‐series data for head yaw angles and steering angles, with the significance level set at *α* = 0.05. Since the data demonstrated a normal distribution, cross‐correlation analysis was then implemented to quantify the temporal relationship between head movements and steering manoeuvres. To interpret the strength of the correlation, we adopted a commonly used classification scale [23] to interpret the relative strength of the correlation coefficients: an absolute value between 0.00 and 0.30 indicates a very weak correlation; 0.31 to 0.50 indicates a weak correlation; 0.51 to 0.70 indicates a moderate correlation; 0.71 to 0.90 indicates a strong correlation; and 0.91 to 1.00 indicates a very strong correlation.

## 3 Results

**FIGURE 4:**
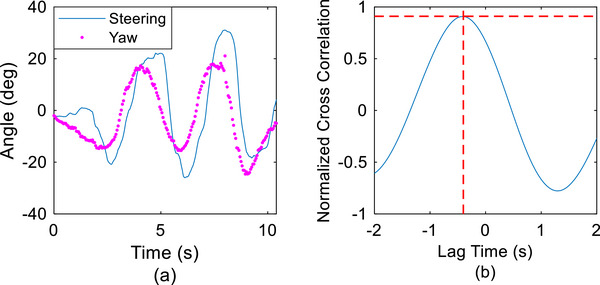
Head‐steering coordination in the slalom driving test. (a) Raw trajectories of the steering and head horizontal (yaw) angles. (b) Cross‐correlogram.

The head‐yaw angle (horizontal rotation around the vertical axis) is related to the head‐turning motion for environmental perception and safety confirmation. The steering angle is related to the user's driving operation for vehicle turning. Figure 4a illustrates a typical example of head movement and steering angle measured in the slalom driving test of the third participant (P3). This result clearly demonstrated the feasibility of the proposed simultaneous recording of these two values. They had a similar trend, but the participants’ head movements varied just before the vehicle steering throughout the process of slalom driving; that is, these two signals had a significant correlation in the temporal relationship.

For quantitatively analysing the temporal relationship between head movements and steering manoeuvres, a cross‐correlogram, a graphical representation of the cross‐correlation function between these two signals in time series, was then examined. As depicted in Figure 4b, the x‐axis represents the time lag, and the y‐axis represents the cross‐correlation value. The time‐series point having the maximum cross‐correlation value indicates that the two time‐series signals were most synchronised at that specific time shift, that is, the time lag between head movements and steering operation. In this trial, the peak value in the cross‐correlogram was 0.91, located at the time point of −0.4 s, indicating that the participant's head movement led his steering operation by 0.4 s during the slalom driving.

**FIGURE 5:**
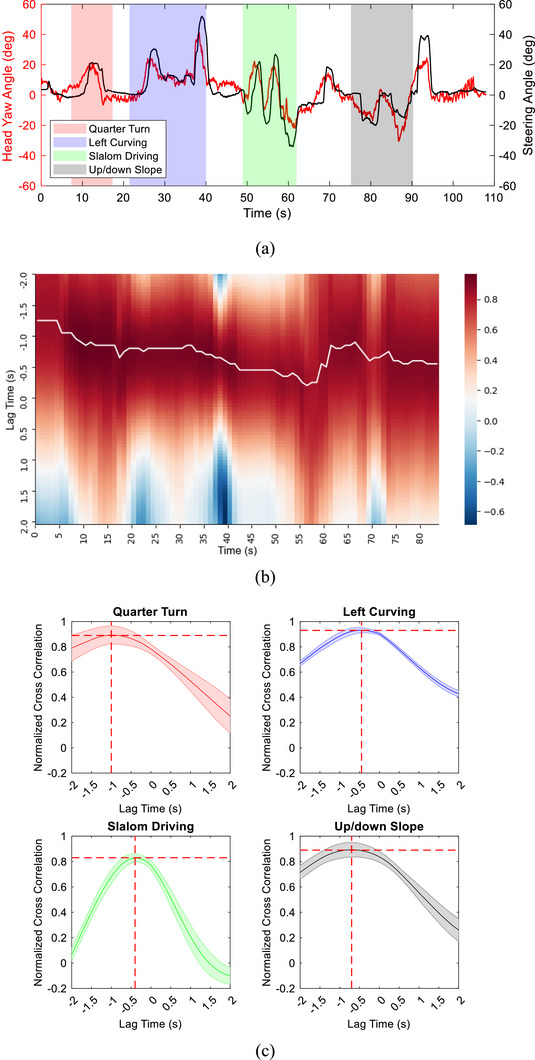
Driving behaviour analysis of P3. (a) Trajectories of head‐yaw angle and steering angle. (b) Rolling windowed cross‐correlogram. The white curve indicates the position of the maximum R. (c) Averaged cross‐correlogram across five trials. The shadowed areas represent one standard deviation from the mean.

To assess the user's behaviour dynamics over a continuous driving period, the participants were instructed to drive the MMS to complete the designated route on the outdoor wheelchair training ground. Figure 5a compares the head‐yaw and steering angles in a driving trial of P3. The overall Pearson correlation coefficient was 0.79 (*p* < 0.0001), demonstrating a strong correlation between head movements and steering manoeuvres according to the reference correlation scale [23]. A rolling window cross‐correlation analysis was conducted to examine the temporal dynamics in greater detail.

As shown in Figure 5b, the white curve depicts the maximum cross‐correlation value in each epoch. The participant's head movements preceded his steering manoeuvres throughout the process, but the time lag varied with respect to the driving courses. The minimum time lag occurred during the 56th epoch, that is, the period ranging from 56 to 76 s, during which the participant drove through the slalom obstacles and then had to turn right immediately. In this scenario, the participant did not have enough time to rotate his head for comprehensive visual perception, resulting in a smaller head‐steering lag.

Figure 5c illustrates the averaged cross‐correlogram of the four designated tasks measured in the trials of P3. For each task, the cross‐correlogram had similar envelopes across all trials, indicated by the minor standard deviations. Furthermore, their maximum cross‐correlation values were high, demonstrating that the participant followed a similar driving pattern and head‐steering coordination.

Table 1 summarises the lag times and cross‐correlation values in the four specified tasks for all three participants. Head‐steering coordination was consistent across all participants; that is, the participants’ head movements led their steering manoeuvres across all task trials. Although the time lag for the same task was slightly varied among participants, the time lags in the continuous turning tasks (left curving and slalom driving) were smaller than in the one‐action turning tasks (quarter turn and up/down slope). Continuous turning likely required persistent focus on the environment perception and head‐steering coordination, increasing the users’ cognitive burden and reducing their reaction time for steering manoeuvres.

**TABLE 1:** Summary of estimated lag times between head movement and steering operation.

Course		Participants			Average (*Mean* ± *SD*)
		P1	P2	P3	
**Quarter Turn**	Lag^a^ [s]	−0.8	−0.6	−1.0	−0.80 ± 0.20
	*r* ^b^	0.90 ± 0.06	0.88 ± 0.06	0.89 ± 0.07	0.89 ± 0.01
**Left Curving**	Lag [s]	−0.4	−0.6	−0.45	−0.48 ± 0.10
	*r*	0.92 ± 0.02	0.89 ± 0.13	0.93 ± 0.02	0.91 ± 0.02
**Slalom Driving**	Lag [s]	−0.6	−0.4	−0.4	−0.47 ± 0.12
	*r*	0.88 ± 0.03	0.84 ± 0.05	0.83 ± 0.33	0.85 ± 0.27
**Up/down Slope**	Lag [s]	−0.8	−1.0	−0.7	−0.83 ± 0.15
	*r*	0.76 ± 0.05	0.81 ± 0.03	0.89 ± 0.06	0.82 ± 0.07

Abbreviations: *r* = cross‐correlation coefficient; SD = standard deviation.

^a^ Negative values indicate that the head movement preceded the steering operation.

^b^ *Mean* ± *SD* across five trials. Positive values indicate that the head movement and the steering operation are in the same direction.

## 4 Discussion

The widespread adoption of MMSs has been accompanied by a concerning rise in accidents, with user behaviour identified as a primary contributing factor. However, conventional assessment methodologies of MMS driving typically depend on qualitative and subjective visual observations by human evaluators [24]. These methods often fail to capture fine‐grained behavioural dynamics, such as the subtle coordination between visual perception and steering manoeuvres. This lack of objective quantification hinders the development of effective safety interventions for older adults and individuals with mobility impairments. Therefore, establishing a quantitative assessment framework is of significant clinical and practical importance to facilitate the early identification of hazardous driving patterns. To address this gap, this study presented the first attempt to incorporate automated behaviour monitoring into the quantitative driving evaluation of MMSs utilising an add‐on driving behaviour monitoring system. The experimental results demonstrated high synchronicity between steering operations and head poses with observable peak time lags. This implies that the developed system successfully captured critical behavioural aspects associated with safety confirmation during turning, providing objective and reproducible insights that mark a methodological leap forward from traditional subjective assessments.

In addition, the cross‐correlation between head movements and steering angles consistently demonstrated stable and repeatable trends across all participants and driving tasks. Although further validation using a larger cohort is warranted, these preliminary results suggest that cross‐correlation analysis could serve as a robust, quantitative index indicative of safe driving practices. In the automotive domain, similar cross‐correlation methods have been effectively employed to quantitatively evaluate driver safety and attentiveness [16]. Consequently, our findings underscore the potential of adopting cross‐correlation methodologies in MMS driving, contributing significantly toward establishing objective metrics for assessing driving behaviours and overall safety.

This study has several limitations that should be addressed in future research. First, the current experimental design was not intended to validate the measurement accuracy of the proposed ADBMS; accuracies of steering and throttle operation angles and head‐pose estimation have already been evaluated in the studies that originally introduced these techniques [19]. Accordingly, the present work focuses on establishing proof of concept. Second, as the Pearson correlation coefficient *r* between head‐yaw and steering angles in Figure 5a was 0.79, the coefficient of determination (*r^2^* = 0.624) suggests that approximately 62.4% of the variance in steering operation can be explained by head movement. This indicates a strong predictive relationship, yet it also implies that other factor, such as environmental constraints, may contribute to the remaining variance in driving behaviour. Future investigations will aim to incorporate environmental perception data (e.g., distance to obstacles) into the analysis. Third, the driving experiment included only able‐bodied participants and did not include elderly individuals. Nevertheless, our main finding—that the proposed system can quantify drivers’ behaviour related to visual confirmation of the surrounding environment preceding turning manoeuvres—was robust across participants and driving tasks. Investigating how this time lag varies with physical and cognitive capabilities will be the focus of future work.

## 5 Conclusion

In this study, we developed an innovative add‐on monitoring system, ADBMS, specifically designed to offer an accessible, cost‐effective, and contactless platform for comprehensively measuring user driving behaviours in MMSs. Experimental findings indicated a robust correlation between head movements and steering manoeuvres among users, thus verifying the effectiveness and practicality of ADBMS for driving performance evaluation. Employing cross‐correlation analysis, we objectively quantified the coordination and temporal dynamics between head movements and steering manoeuvres, significantly contributing to our understanding of critical behavioural patterns linked to driving safety. This comprehensive behavioural monitoring facilitates early identification of potentially hazardous driving behaviours, thereby reducing accident risks. Future studies will extend these quantitative metrics by integrating real‐time risk detection systems, aiming to proactively enhance MMS operational safety through timely interventions and alerts.

## Author Contributions

J. S. and T. I. proposed the concept and methodology. Y. L. conceived and conducted the experiments. Y. L. and J. S. performed statistical analysis and figure generation. Y. L. wrote the original draft; T. I. and J. S. reviewed and edited the draft.

## Funding

This work was supported in part by the Japan Society for the Promotion of Science, Grant‐in‐Aid for Scientific Research (KAKENHI), under Grants 23K25253 and 23K17265, and by Fundamental Research Funds for the Central Universities, No. 3072024WD0704 and 3072024CFJ0704.

## Ethics Statement

The experimental procedures were approved by the Institutional Review Board of the National Rehabilitation Center for Persons with Disabilities (protocol number: 2023–074), and all methods were performed in accordance with the relevant guidelines and regulations. All participants provided written informed consent.

## Conflicts of Interest

The authors declare no conflicts of interest.

## Data Availability

The data that support the findings of this study are available upon reasonable request to the corresponding author.
